# Comparison of the effect of cardioplegic solutions used in cardiac surgery on myocardial protection

**DOI:** 10.1590/1806-9282.20241451

**Published:** 2025-05-02

**Authors:** Mahmut Padak, Reşat Dikme, Ezhar Ersöz, Yasemin Hacanlı

**Affiliations:** 1 Harran University, Faculty of Health Sciences, Department of Perfusion – Şanlıurfa, Turkey.; 2 Harran University, Vocational School of Health Services, Dialysis Programme – Şanlıurfa, Turkey.; 3 Harran University, Research and Application Hospital, Department of Perfusion – Şanlıurfa, Turkey.

**Keywords:** Cardiopulmonary bypass, Cardioplegia, Myocardial ischemia, Del Nido

## Abstract

**OBJECTIVE:**

The aim of this retrospective study was to evaluate the effects of cardioplegic solutions on myocardial protection factors in patients undergoing cardiac surgery with cardiopulmonary bypass and to evaluate the relationship between these effects and early clinical outcomes.

**METHODS:**

A total of 78 patients were included in the study. The data of patients who received del Nido cardioplegia (Group 1; n=39) and blood cardioplegia (Group 2; n=39) during the operation were grouped and compared. Preoperative and postoperative routine parameters such as creatine phosphokinase, troponin I, C-reactive protein, and demographic data were recorded, and statistical analyses were performed.

**RESULTS:**

Perfusion time, cross-clamp time, act pump, preoperative C-reactive protein, preoperative creatine phosphokinase, preoperative troponin I, postoperative C-reactive protein, postoperative creatine phosphokinase, postoperative troponin I values were significantly different between del Nido and blood groups (p<0.05).

**CONCLUSION:**

Del Nido cardioplegia provided myocardial protection and early postoperative favorable results compared to blood cardioplegia, which may make it a viable option for conventional cardiopulmonary bypass.

## INTRODUCTION

The heart is the largest producer and consumer of energy compared to other organs in our body. It mainly oxidizes fatty acids and carbohydrates to produce energy in the form of adenosine triphosphate (ATP) and other high-energy phosphates. It has the potential to produce approximately 35 times its own weight in ATP in a day^1^. Therefore, a continuous supply of oxygen to the heart is required to maintain its contractile function. The interruption of blood flow to the heart is termed "ischemia," and this condition develops due to the interruption of both oxygen and substrate supply^2^.

Myocardial protection is important during cardiac surgery. In cardiac surgery with cardiopulmonary bypass, the heart is usually stopped with cardioplegic solutions^3^. Stopping the heart in diastole with hyperkalemic solutions reduces myocardial oxygen consumption and allows the surgeon to work in an immobile and bloodless field. Cardioplegia solutions commonly used in adult cardiac surgery are multidose blood and single-dose del Nido cardioplegia solution (dNCS)^4^.

Del Nido cardioplegia (DNC) is an extracellular solution developed by Pedro del Nido and his team at the University of Pittsburgh in the early 1990s^5^. dNCS was initially designed to enhance myocardial protection of immature cardiomyocytes and has been used in clinical practice for pediatric and infant patients. In recent years, it has been widely used in congenital heart surgery and has provided a well-tolerated myocardial protection time of over 90 min^6^. Aspects of dNCS use include single-dose administration, shorter aortic cross-clamp (AoX) and cardiopulmonary bypass (CPB) times, less need for defibrillations after AoX, and less release of postoperative cardiac enzymes^7^. dNCS is a crystalloid-based solution with approximately 75% less calcium (Ca2+) and contains magnesium sulfate and lidocaine, which limit intracellular Ca^2+^ accumulation and promote ventricular recovery^8^.

The choice of cardioplegic solution in cardiac surgery depends on several factors, including the type of surgery and the surgeon's preference and expertise. Although blood cardioplegia is a traditional choice and continues to be widely used, dNCS is gaining popularity in adult cardiac surgery following its proven safety in pediatric cardiac procedures^9^.. Although some congenital or acquired heart diseases are successfully treated, open heart surgery is sometimes required. Although cardioplegia is currently used in patients undergoing cardiac surgery, the ideal cardioplegic technique remains controversial. There is no consensus on best practices for myocardial protection assessment. First, there is no way to routinely perform real-time myocardial protection assessment. Second, myocardial protection is clinically assessed through a number of indirect factors, such as postoperative troponin I/T or creatine kinase MB levels, ischemic electrical signs on ECG or myocardial infarction, stroke, atrial fibrillation, myocardial function on echo, low cardiac output status, inotropic support, intra-aortic balloon pump, extracorporeal membrane oxygenation, as well as time to extubation and ICU stay^10^.

In our study, we aimed to investigate the effects of del Nido and blood cardioplegia on myocardial protection in the postoperative period in patients who underwent cardiac surgery with cardiopulmonary bypass.

## METHODS

This retrospective study used the data of patients who underwent cardiac surgery with cardiopulmonary bypass between January 2021 and December 2023 in the Cardiovascular Surgery Clinic of Harran University Hospital. It was approved by the Harran University Faculty of Medicine Clinical Research Ethics Committee with the decision dated 27.05.2024, session 07, session 03. This study was conducted in accordance with the Declaration of Helsinki revised in 1989.

### Data collection method

Data of the patients were obtained from computers, operating theater records, perfusion follow-up records, intensive care follow-up cards, and file records. The data of the patients obtained in this study were recorded and entered into the computer.

### Statistical analysis

SPSS 25.0 (IBM, Chicago, IL, USA) software was used for data analysis and statistical evaluations. "Mann-Whitney U Test" was used for comparison and significance between two independent groups. Spearman's correlation analysis was used to examine the relationships within and between different groups. Data were presented as mean±standard deviation for numerical variables and number and percentage for categorical variables. A two-tailed p-value <0.05 was accepted as an indicator of a statistically significant difference.

## RESULTS

According to Mann-Whitney U test, there was a significant difference between del Nido and blood groups in perfusion time, cross-clamp time, act pump, preoperative CRP, preoperative CK-MB, preoperative troponin I, postoperative CRP, postoperative CK-MB, and postoperative troponin I values (p<0.05) (Table 1).

**Table 1:** Descriptive statistics for del Nido and blood cardioplegia group.

Del Nido cardioplegia group				
	Minimum	Maximum	Mean	Sth. deviation
Age	34	79	52.23	10.289
Height	160	185	168.67	6.662
Weight	52	86	75.28	8.778
BSA	1.53	2.04	1.8433	0.12158
Flow	3,660	4,890	4,448.21	282.134
Perfusion time	51	221	124.05	36.687
Cross-clamp time	37	150	84.74	27.776
ACT pump	420	1,000	617.51	164.643
Preoperative CRP (mg/L)	0.05	37.37	3.5149	7.29675
Preoperative CK-MB (u/L)	0.39	69.54	27.2300	28.28622
Preoperative troponin I (ng/mL)	0.00	14,854.20	5,492.0910	6,116.40436
Postoperative CRP (mg/L)	0.10	130.18	17.5105	28.36096
Postoperative CK-MB (u/L)	0.20	9.13	2.1349	1.80895
Postoperative troponin I (ng/mL)	0.03	51.43	21.8670	20.34364
**Blood cardioplegia group**				
Age	22	76	59.95	10.224
Height	150	187	169.00	8.672
Weight	55	133	76.62	15.786
BSA	1.56	2.55	1.8715	0.21606
Flow	3,750	6,110	4,500.56	519.402
Perfusion time	60	249	105.92	39.783
Cross-clamp time	33	173	71.56	34.776
ACT pump	414	1,145	646.23	189.534
Preoperative CRP (mg/L)	0.16	126.82	14.7038	25.04534
Preoperative CK-MB (U/L)	0.59	189.58	21.6041	50.28994
Preoperative troponin I (ng/mL)	0.00	12,978.44	2,107.5348	4,558.43031
Postoperative CRP (mg/L)	0.29	194.81	78.6500	44.12288
Postoperative CK-MB (u/L)	0.00	150.10	11.6608	26.46399

ACT: activated clotting time; BSA: body surface area; CRP: C-reactive protein; CK-MB: creatine phosphokinase.

According to the table, in the del Nido patients group, a negative significant correlation between height and preoperative CK-MB (r: −0.362, p: 0.024), a negative significant correlation between height and preoperative troponin I (r: −0.336, p: 0.037), a negative significant correlation between height and postoperative CK-MB (r: −0.448, p: 0.004), a negative significant correlation between BSA and preoperative CK-MB (r: −0.427, p: 0.007), a negative significant correlation between act pump and preoperative CK-MB (r: −0.445, p: 0.004), a negative significant correlation between act pump and preoperative troponin I (r: −0.319, p: 0.048), a positive significant correlation between act pump and postoperative CRP (r: 0.426, p: 0.007), a positive significant correlation between preoperative CRP and postoperative CK-MB (r: 0.399, p: 0.012), a positive significant correlation between preoperative CK-MB and preoperative troponin I (r: 0.839, p: 0.000), and a negative significant correlation between preoperative CK-MB and postoperative CRP (r: −0.326, p: 0.043) were observed.

According to Table 2, there was a positive significant correlation between preoperative CRP and postoperative CRP in the blood group (r: 0.408, p: 0.010), a positive significant correlation between preoperative CRP and postoperative CK-MB (r: 0345, p: 0,031), a positive significant correlation between preoperative CRP and postoperative troponin I (r: 0.394, p: 0,013), a positive significant correlation between preoperative CK-MB and postoperative troponin I (r: 0.774, p: 0.000), a positive significant correlation between preoperative troponin I and postoperative troponin I (r: 0.628, p: 0.000), and a positive significant correlation between postoperative CK-MB and postoperative troponin I (r: 0.620, p:0,000).

**Table 2:** Correlation table of del Nido group and blood group.

Del Nido cardioplegia group		Preoperative CK-MB (U/L)	Preoperative troponin I (ng/mL)	Postoperative CRP (mg/L)	Postoperative CK-MB (u/L)
Height	r	-0.362*	-0.336*		-0.448**
	p	0.024	0.037		0.004
BSA	r	-0.427**			
	p	0.007			
ACT pump	r	-0.445**	-0.319*	0.426**	
	p	0.004	0.048	0.007	
Preoperative CRP (mg/L)	r				0.399*
	p				0.012
Preoperative CK-MB (U/L)	r		0.839**	-0.326*	
	p		0.000	0.043	
**Blood cardioplegia group**		**Preoperative troponin I**	**Postoperative CRP (mg/L)**	**Postoperative** **CK-MB (u/l)**	**Postoperative troponin I (ng/mL)**
Preoperative CRP (mg/L)	r		0.408**	0.345*	0.394*
	p		0.010	0.031	0.013
Preoperative CK-MB (U/L)	r	0.774**			
	p	0.000			
Preoperative troponin I (ng/mL)	r				0.628**
	p				0.000
Postoperative CK-MB (u/L)	r				0.620**
	p				0.000

ACT: activated clotting time, BSA: body surface area, CRP: C-reactive protein, CK-MB: creatine phosphokinase.

* Correlation is significant at the 0.05 level.

** Correlation is significant at the 0.01 level.

In the correlation of the two groups, a negative significant correlation (r: −0,248 p: 0,028) was found between perfusion time and preoperative CRP, a positive significant correlation between perfusion time and preoperative troponin I (r: 0.262, p: 0.021), a negative significant correlation between perfusion time and preoperative CRP (r: −0.228, p: 0.003), a negative significant correlation between cross-clamp time and preoperative CRP (r: −0.279, p: 0.013), a negative significant correlation between cross-clamp time and postoperative CRP (r: −0.344, p: 0.002), a negative significant correlation (r: −0.269, p: 0.017) was found between act pump and preoperative CK-MB, a negative significant correlation (r: −0.288, p: 0.011) was found between preoperative CRP and preoperative CK-MB, a negative significant correlation was found between preoperative CRP and preoperative troponin I (r: −0.398, p: 0,000), and a positive significant correlation between preoperative CRP and postoperative CRP (r: 0.677, p:0.000).

According to Table 3, there was a positive significant correlation between preoperative CRP and postoperative CK-MB (r: 0.555, p: 0.000), a positive significant correlation between preoperative CK-MB and preoperative troponin I (r: 0.764, p: 0.000), a negative significant correlation between preoperative CK-MB and postoperative CRP (r: −0.402, p: 0.000), a positive significant correlation between preoperative CK-MB and preoperative troponin I (r: 0.249, p: 0.028), a negative significant correlation between preoperative troponin I and postoperative CRP (r: −0.537, p: 0.000), a positive significant correlation between preoperative troponin I and postoperative troponin I (r: 0.647, p: 0.000), a positive significant correlation between postoperative CRP and postoperative CK-MB (r: 0.421, p: 0.000), a negative correlation between postoperative CRP and postoperative troponin I (r: −0.267, p: 0.018).

**Table 3:** Correlation table in del Nido and blood group.

Del Nido and blood group		Preoperative CRP (mg/L)	Preoperative CK-MB (U/L)	Preoperative troponin I (ng/mL)	Postoperative CRP (mg/L)	Postoperative CK-MB (U/L)	Postoperative troponin I (ng/mL)
Perfusion time	r	-0.248*		0.262*	-0.328**		
	p	0.028		0.021	0.003		
Cross-clamp time	r	-0.279*			-0.344**		
	p	0.013			0.002		
ACT pump	r		-0.269*				
	p		0.017				
Preoperative CRP (mg/L)	r		-0.288*	-0.398**	0.677**	0.555**	
	p		0.011	0.000	0.000	0.000	
Preoperative CK-MB (U/L)	r			0.764**	-0.402**		0.249*
	p			0.000	0.000		0.028
Preoperative troponin I (ng/mL)	r				-0.537**		0.647**
	p				0.000		0.000
Postoperative CRP (mg/L)	r					0.421**	-0.267*
	p					0.000	0.018

ACT: activated clotting time, CRP: C-reactive protein, CK-MB: Creatine phosphokinase.

* Correlation is significant at the 0.05 level.

** Correlation is significant at the 0.01 level.

## DISCUSSION

Before heart surgery, it is necessary to stop the heart quickly. In order to better restore the function of the myocardium after ischemia, the concept of cardioplegia has been proposed. With the development of cardiac surgery, myocardial protection research has been going on for decades, and the establishment of the general theoretical system of myocardial protection and clinical application methods are becoming more and more mature^11^.

DNC is a type of single-dose cardioplegia. The initial infusion dose is usually 20 mL/kg and can be up to a maximum of 1 L, which can meet the cross-clamp time of 90 min. If the duration of cardiac arrest needs to be extended, the amount of intraoperative infusion is usually added at a dose of 10 mL/kg. The results of various studies have shown that the perfusion volume of DNC is significantly lower than that of blood-containing cardioplegia, requiring repeated perfusion^12^.

Troponin is released from damaged cell membranes only after cardiac muscle cells have died, and both cardiac troponin values are ideal for detecting myocardial injury. CK-MB is an important biomarker in the diagnosis of myocardial injury and is used as a potential co-detection index along with troponin to reflect the degree of myocardial injury^13^. DNC was associated with lower troponin and CK-MB after cardiac surgery in a meta-study including only two randomized clinical trial (RCT) studies. A recent meta-analysis of del Nido cardioplegia for myocardial protection included three RCTs and showed no statistically significant difference in troponin levels 24 h after cardiac surgery between the DNC group and the CBC group. Among the 10 RCTs included in this study, there were three, four, and five studies with complete data including troponin I and CK-MB levels 24 h after surgery, respectively. The results showed that DNC significantly reduced troponin and CK-MB levels compared to blood cardioplegia. No significant difference was found in postoperative troponin I values^14^.

In one study, DNC showed potential superiority in myocardial protection, as evidenced by detailed evaluation of both functional and biochemical markers. Postoperative LV ejection fraction changes, which serve as a functional indicator of myocardial protection, showed comparable results between the two groups, while the del Nido group outperformed the blood cardioplegia group in terms of changes in troponin I level, an important biochemical marker of myocardial protection^15^. In our study, the mean values of perfusion time, cross-clamp time, preoperative CK-MB, preoperative troponin I, and postoperative troponin I were higher in the dNCS group, while the mean values of preoperative CRP, postoperative CRP, and postoperative CK-MB were higher in the blood cardioplegia group. According to these results, the duration of operation, aortic cross-clamping time, and postoperative troponin I values were higher in the dNCS group, while postoperative CRP and CK-MB values were higher in the blood group.

## CONCLUSION

In our study, preoperative troponin I value was in the same direction with preoperative CK-MB and perfusion time. Preoperative and postoperative CRP values were in the same direction with postoperative CK-MB values.
